# Supplementary material for: Cloning of the broadly effective wheat leaf rust resistance gene Lr42 transferred from Aegilops tauschii
Source: Nat Commun. 2022 Jun 1;13:3044. doi: 10.1038/s41467-022-30784-9 (PMC9160033; doi:10.1038/s41467-022-30784-9)
Supplement: Supplementary file 1 — Supplementary Information [file 41467_2022_30784_MOESM1_ESM.docx]

**Cloning of the broadly effective wheat leaf rust resistance gene *Lr42* transferred from *Aegilops tauschii***

Lin *et al*.

**
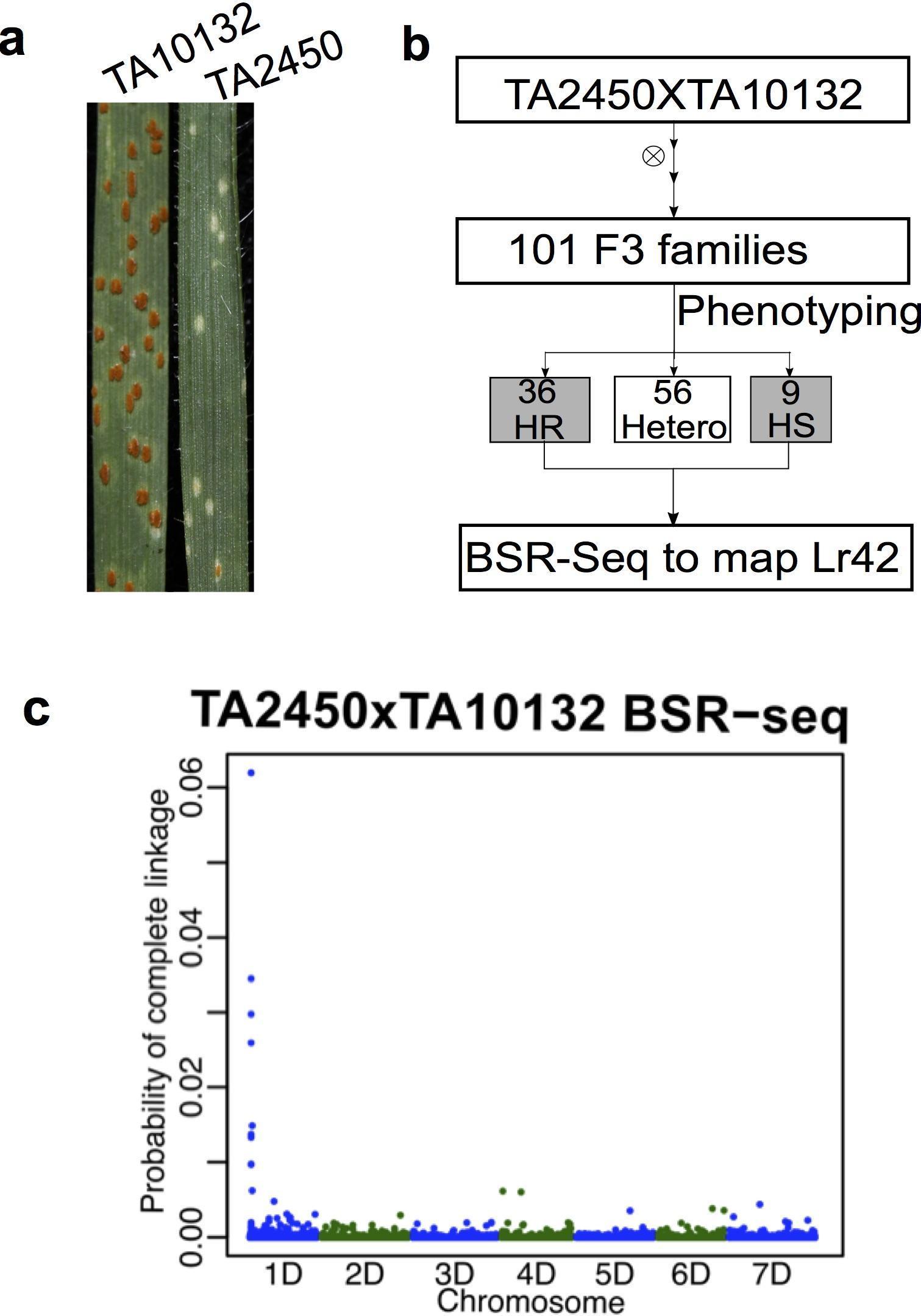
**

**Supplementary Fig. 1: Genetic mapping of the *Lr42* gene with population TA2450 x TA10132.** (**a**) Phenotype of *Ae. tauschii* accession TA10132 (*lr42*) was susceptible (Infection Type = 33+) and phenotype of TA2450 (*Lr42*) was hypersensitive flecks (Infection Type = ; to ;1-) at the seedling stage upon inoculation with race PNMRJ. (**b,c**) Genetic mapping of the *Lr42* gene via BSR-seq. Bi-parental population of TA2450 x TA10132 with 101 F_2:3_ families (15 individuals for each family) were phenotyped, and segregated with 36 homozygous resistant (HR) families, 9 homozygous susceptible (HS) families, and 56 heterozygous families. The reason for segregation distortion at the *Lr42* locus in this population is unknown. Among them 26 HR and 9 HS were selected for BSR-seq sequencing. Source data are provided as a Source Data file.


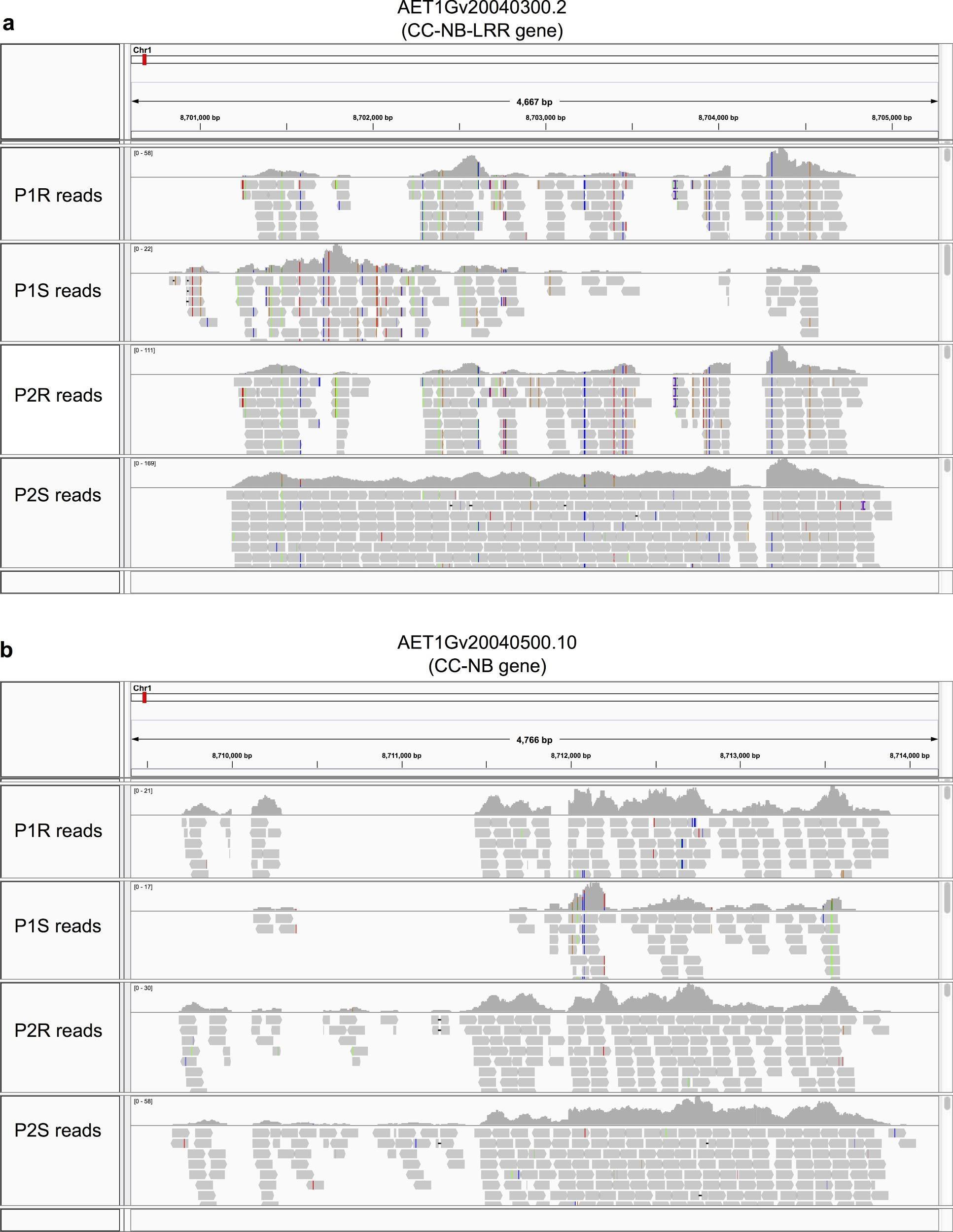


**Supplementary Fig. 2: RNA-Seq reads alignments of the two NLR genes in the mapping interval.** (**a**) Read alignments on gene AET1Gv20040300.2. (**b**) Read alignments on gene AET1Gv20040500.10. Integrative Genomics Viewer (IGV) was used to display alignments. In each panel, the top curve is the distribution of read depth along the gene. Non-gray vertical lines indicate proportions of alleles at a position that contains multiple types of sequences (alleles). Alignments of reads (horizontal bars) are shown under the curve. P1R and P1S represent RNA-Seq data sets of the resistant pool and the susceptible pool from the mapping population 1, respectively. P2R and P2S represent RNA-Seq data sets of the resistant pool and the susceptible pool from the mapping population 2, respectively. Colored lines in reads highlight polymorphisms between reads and the reference genome.

**
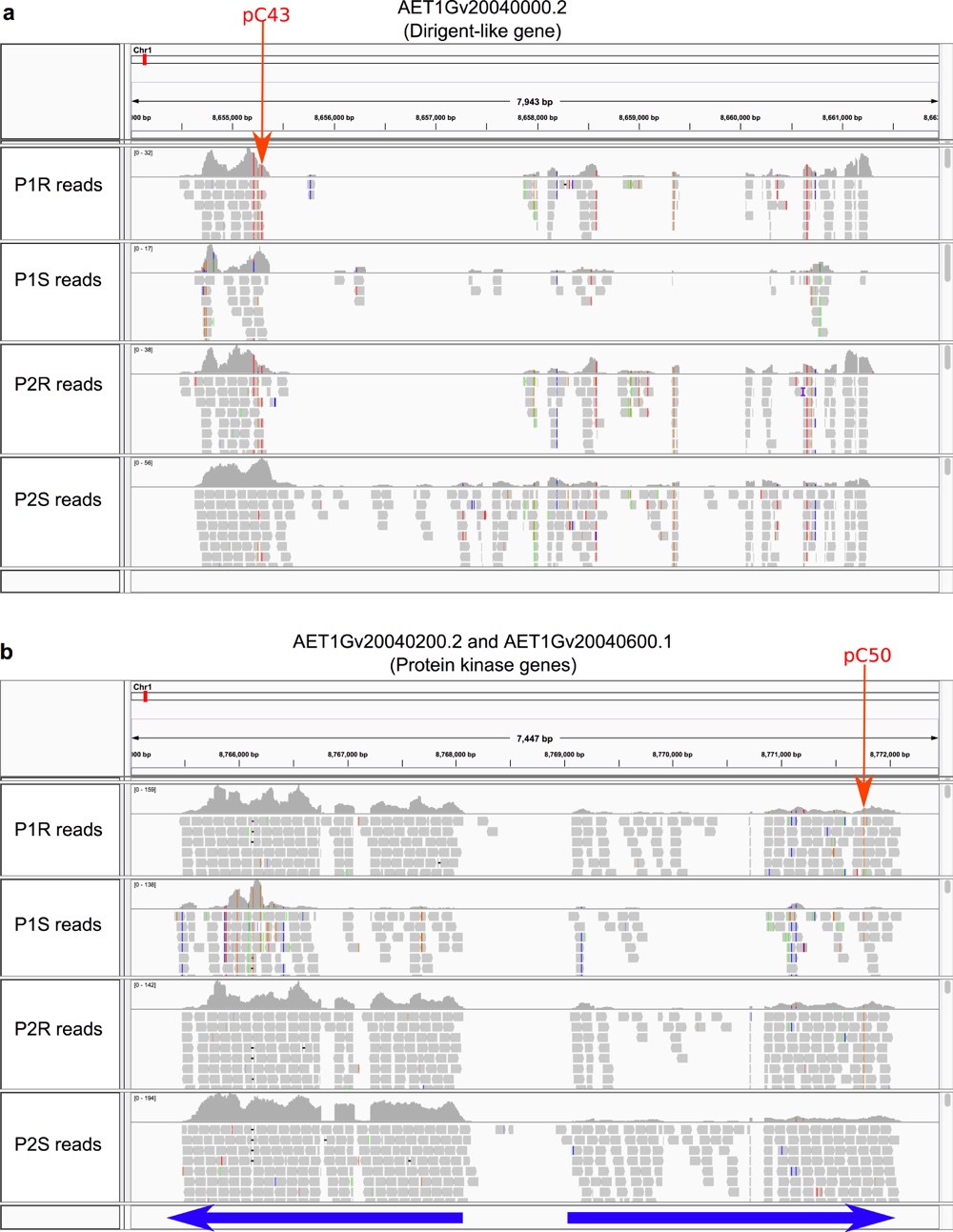
**

**Supplementary Fig. 3: RNA-Seq reads alignments of genes with flanking markers**. Integrative Genomics Integrative Genomics Viewer (IGV) was used to display alignments. In each panel, the top curve is the distribution of read depth along the gene. Non-gray vertical lines represent proportions of alleles at a position that contains multiple types of sequences (alleles). Alignments of reads (horizontal bars) are shown under the curve. P1R and P1S represent RNA-Seq data sets of the resistant pool and the susceptible pool from the mapping population 1, respectively. P2R and P2S represent RNA-Seq data sets of the resistant pool and the susceptible pool from the mapping population 2, respectively. Colored lines in reads highlight polymorphisms between reads and the reference genome. (**a**) Read alignment of the gene AET1Gv20040000.2. (**b**) Read alignment of genes AET1Gv20040200.2 (the first blue arrow) and AET1Gv20040600.1 (the second blue arrow). The KASP markers pC43 and pC50 were indicated with red arrows.


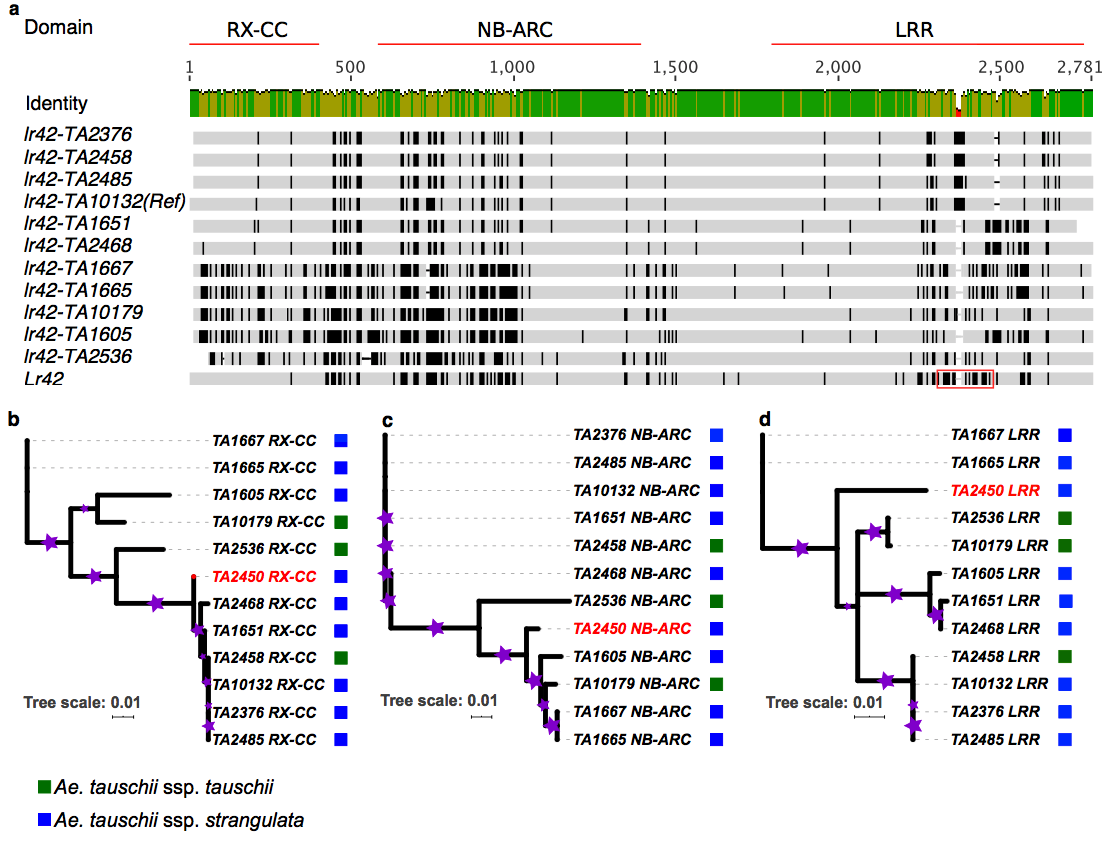


**Supplementary Fig. 4: Sequence and phylogenetic analysis of *Lr42* alleles.** (**a**) Multiple alignments using coding sequences of the 12 *Lr42* alleles were generated with ClustalW in Geneious. Three NLR domains are indicated. The identity track has the colors green, yellow and red, representing identities from high to low. Vertical black lines highlight polymorphisms with the consensus sequence. The red box highlights the unique region (*Lr42*-unique-segment) carried by the *Lr42* allele. (**b-d**) Separate phylogenetic trees using sequences of the RX-CC, NB-ARC and LRR domains. Green and blue squares at tips represent ssp. *strangulata* (L2) and *tauschii* (L1), respectively. The bootstrap values of clades represented by purple stars were size-coded. The range of sizes from small to large corresponds to the bootstrap values from 54% to 100%.


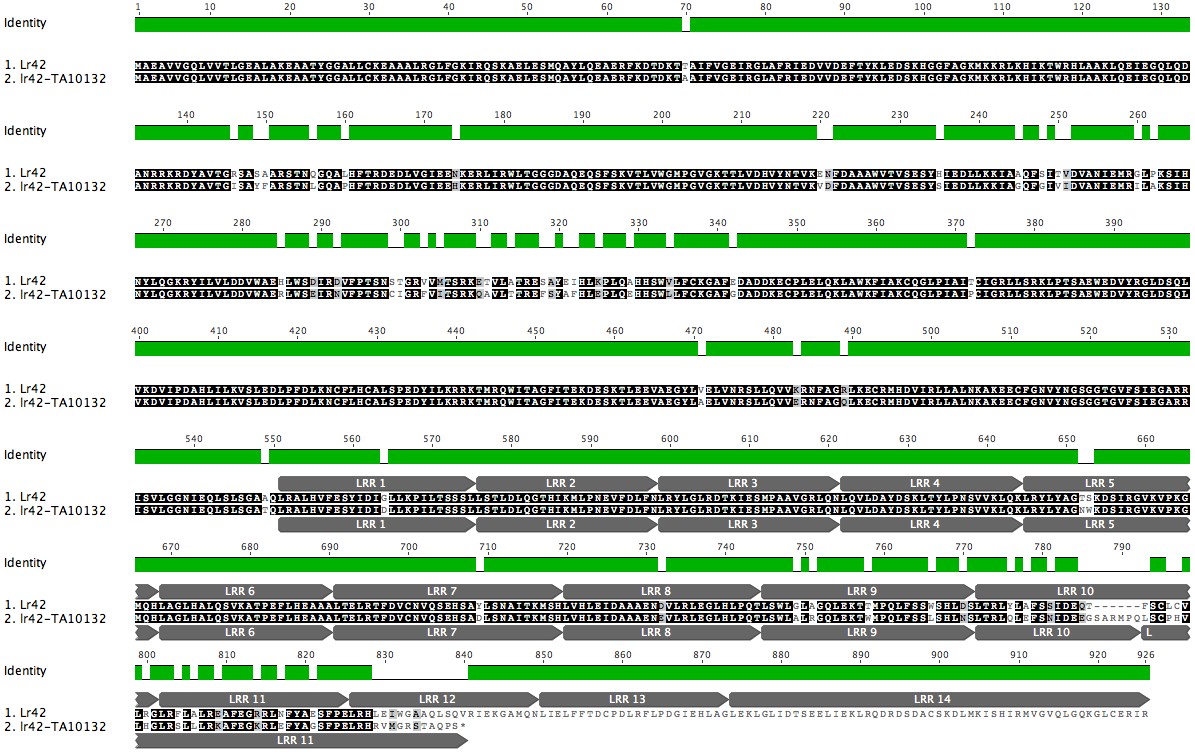


**Supplementary Fig. 5: Protein alignment of Lr42 and lr42-TA10132.** The conserved amino acid residues between two sequences were indicated with black boxes. Fourteen LRR repeats in Lr42 and 11 LRR repeats in lr42-TA10132 were identified. The LRR repeats were labeled with dark gray arrows.

**Supplementary Fig. 6: Histochemical GUS assay and RT-PCR analysis for transient *GUS* expression driven by the *Lr42* native promoter in wheat leaf.** (**a**) GUS expression in the epidermal cells of a wheat leaf at 48 h after agroinfiltration with *Lr42_p_::GUS*. Scale bar = 20 µm. (**b**) *GUS* expression driven by the *Lr42* native promoter was detected in the infiltrated wheat leaves by RT-PCR. The *18S* rRNA gene was used as an internal control. NTC, no template control. M, DNA Ladder. Experiments were repeated twice with the consistent results.


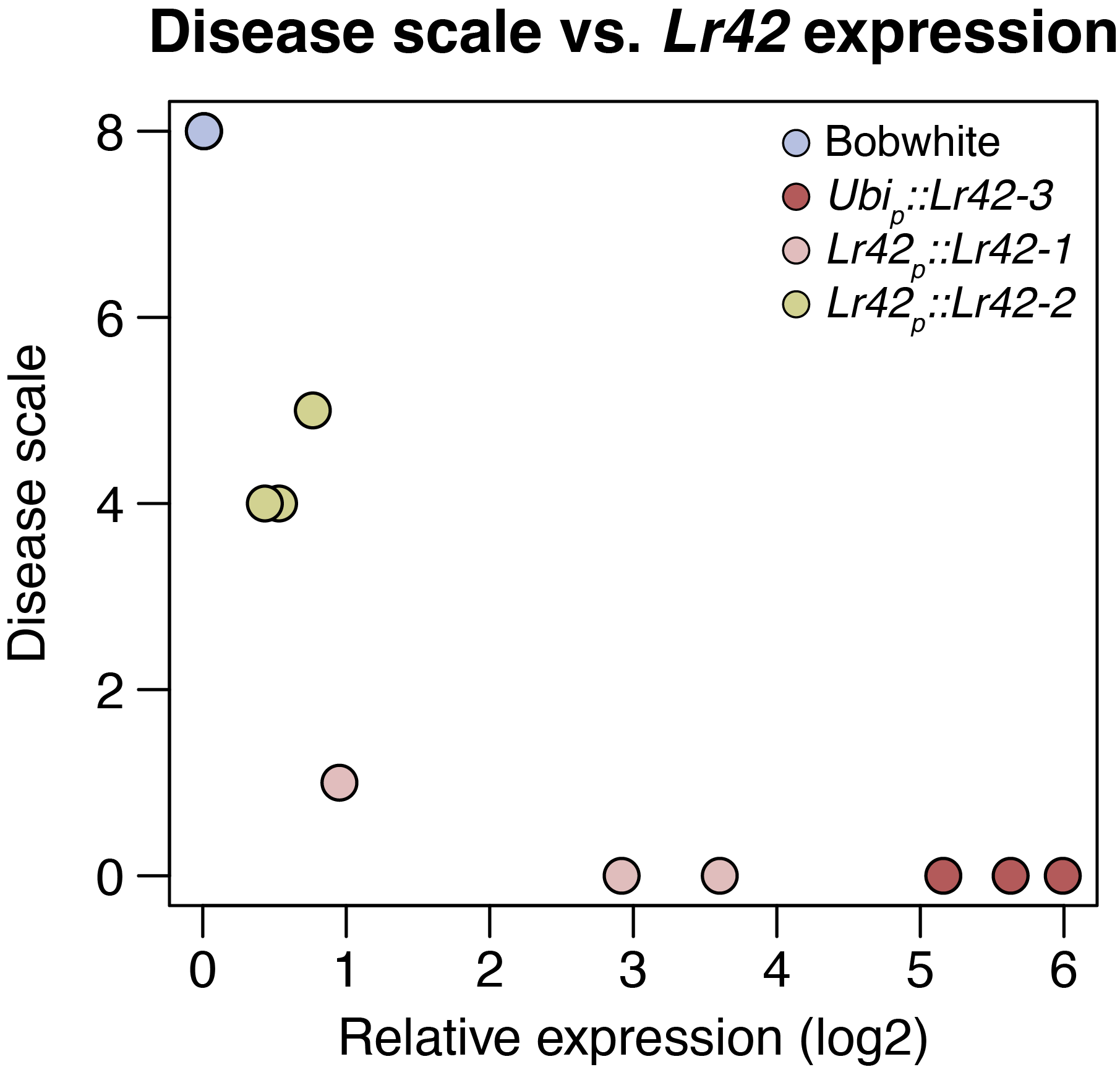


**Supplementary Fig. 7: Disease resistance versus *Lr42* expression.** Original seedling infection types were converted to a 0–9 disease scale (Methods). Relative expression data of *Lr42* were the same as qRT-PCR data used in Fig. 4b. Expression data were transformed with the logarithm of base 2 (log2). Source data are provided as a Source Data file.

**Supplementary Fig. 8: Genomic copy number estimation of *Lr42* via quantitative PCR.** The *Lr42* copy of Thatcher-*Lr42* was normalized to 1. The copy number relative to Thatcher-*Lr42*, carrying homozygous *Lr42*, in each other plant was quantified. Bar heights are means and error bars stand for standard deviations (SD) from two technical replicates per line. Dots represent data from technical replicates. As expected, no copy was detected in either Thatcher or Bobwhite. Copy number variation was evidenced among individuals within the events of *Ubi_p_*::*Lr42-3* (T2 plants) and *Lr42_p_*::*Lr42-2* (T1 plants). Individuals of *Lr42_p_*::*Lr42-1* are also T1 transgenic plants. The infection type (IT) of each individual plant was labeled. Source data are provided as a Source Data file.


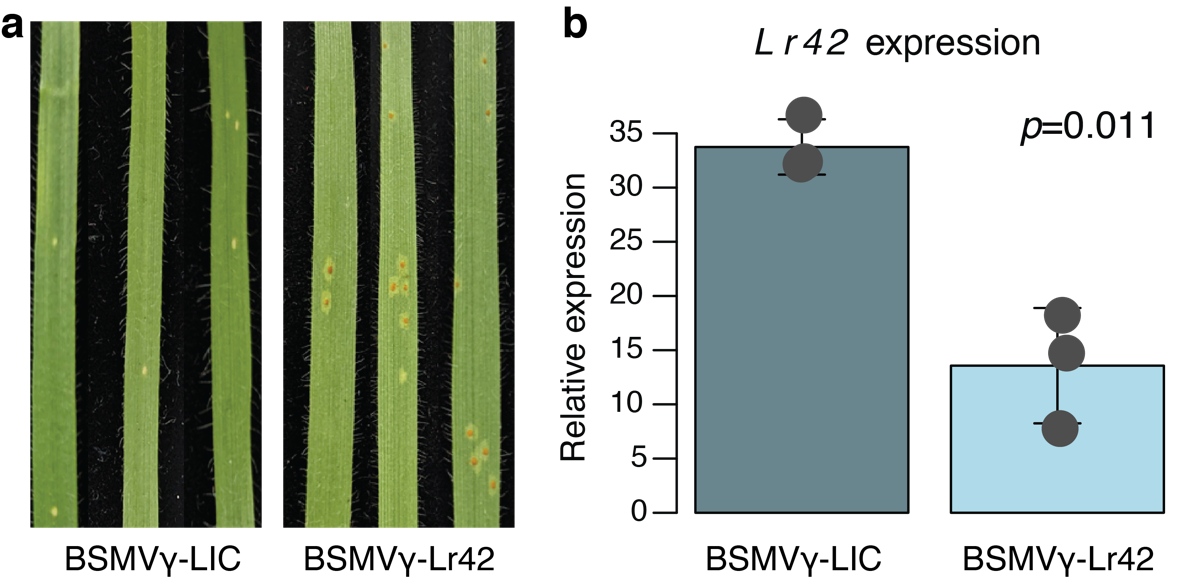


**Supplementary Fig. 9: Validation of *Lr42* mediated resistance by VIGS.** (**a**) Phenotype of leaves infected with the virus containing empty vector (BSMVα, BSMVβ, and BSMVγ-LIC, represented by BSMVγ-LIC) or the *Lr42* fragment (BSMVα, BSMVβ, and BSMVγ-Lr42, represented by BSMVγ-Lr42). (**b**) Expression levels of *Lr42* in BSMVγ-LIC and BSMVγ-Lr42 plants measured by qRT-PCR. Bar heights are means and error bars stand for standard deviations (SD). One plant for each biological replicate, and three biological replicates were used for each group. The p-value (*p*) was from a two-side t-test between the two groups. Source data are provided as a Source Data file.


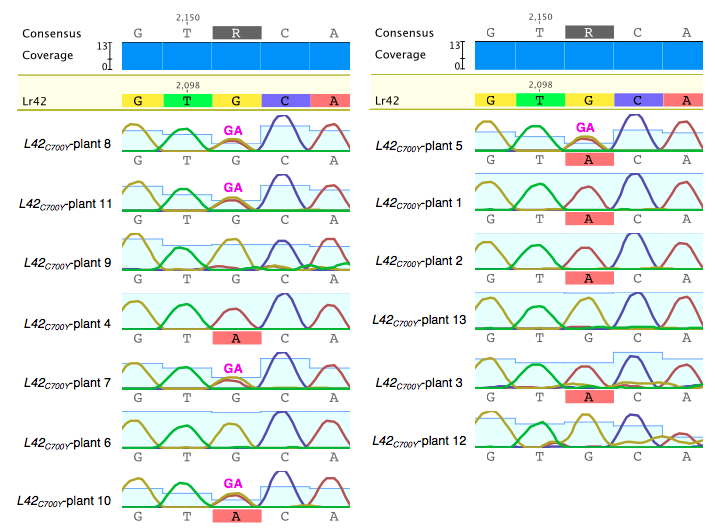


**Supplementary Fig. 10: Sanger sequencing of the mutation site of individuals from the EMS mutant family.** Individual amplicons from the mutation site, C700Y, were sequenced for individuals of the mutant family. “GA” was manually added to represent a heterozygous genotype at the mutation site.


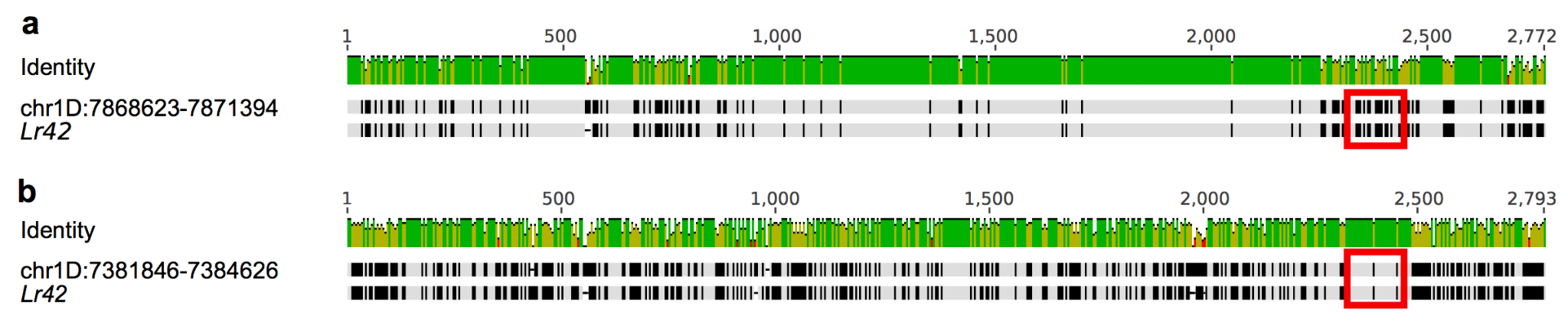


**Supplementary Fig. 11: Pairwise comparison between *Lr42* and a homolog in bread wheat.** Pairwise alignment was performed using the Geneious software. The identity track has the colors green, yellow and red, representing the identities from high to low. Vertical black lines represent polymorphisms between a sequence and the consensus sequence. (**a**) Alignment of the *Lr42* allele with the homolog with the highest similarity (the allelic homolog) in the reference genome of CS. The identity between *Lr42* and the allelic homolog was 93.9%. (**b**) The alignment of the *Lr42* allele with the CS homolog that carries a highly identical sequence with the *Lr42*-unique-segment (135/137 bp match from 2,308 to 2,444 bp at the *Lr42* allele, highlighted with red rectangles). The identity between *Lr42* and this non-allelic homolog was 83.8%.


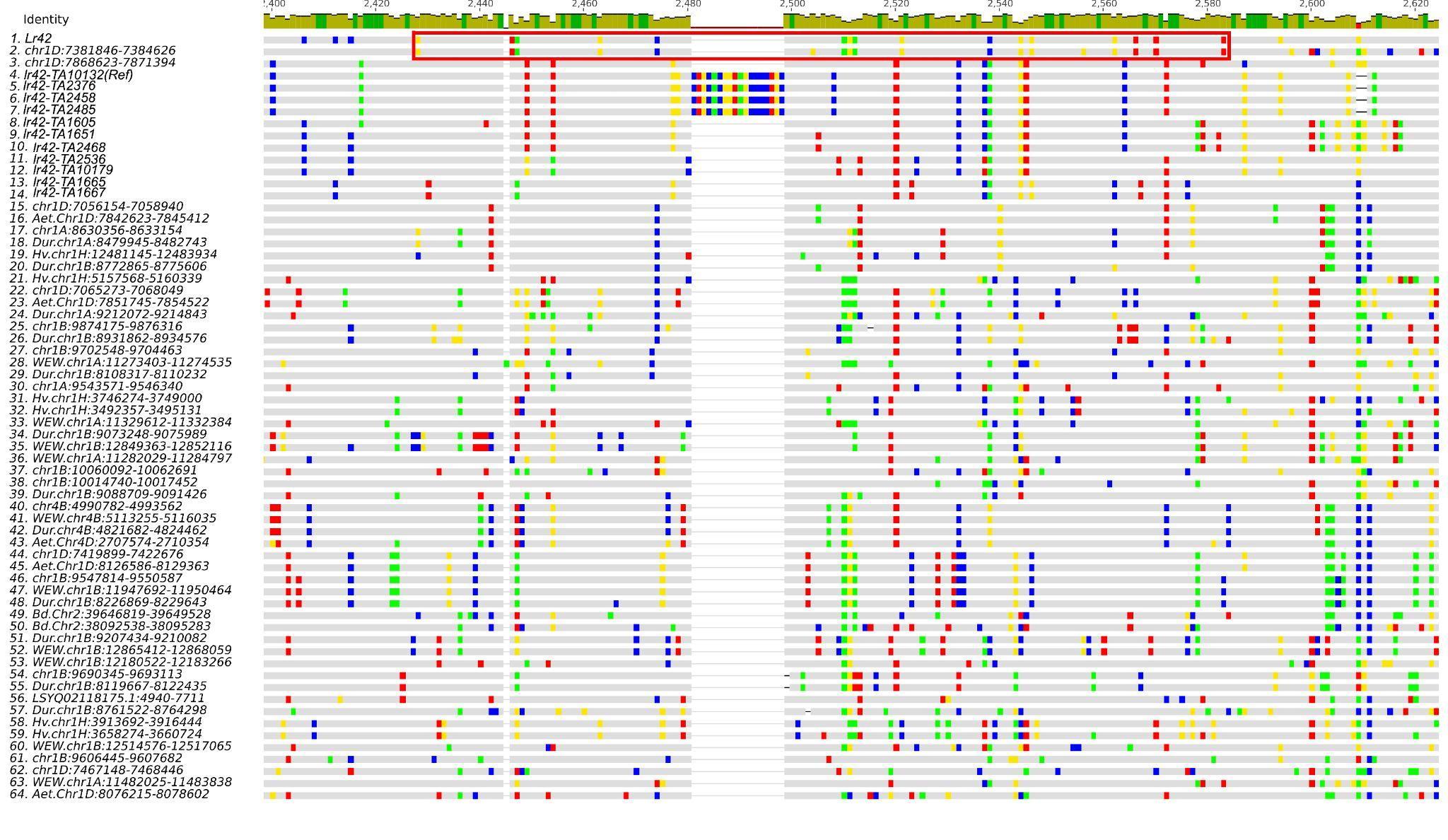
**Supplementary Fig. 12: Multiple alignments of *Lr42* homologs in an LRR region.** The *Lr42* allele was aligned to reference genomes of multiple wheat closely related species using “blastn”, and homologs with at least 1 kb matches were extracted. Multiple alignments of sequences of all these homologs and *Lr42* alleles from *Ae. tauschii* were performed using ClustalW in Geneious. The sequences that did not contain a region matching the *Lr42*-unique-segment (highlighted with red rectangle) were manually removed. Green, yellow and red colors of the “identity” track represent the identities of high, medium, and low, respectively. Colors on sequences highlight polymorphisms between a sequence and the consensus sequence. The Sequence IDs beginning with “Aet” are from the reference genome of *Ae. tauschii* (Aet v4.0); sequence IDs beginning with “chr” are from the reference genome *T. aestivum* cv. CS (iwgsc_refseqv1.0); sequence IDs beginning with “Dur” are from the reference genome of *T. turgidum* subsp. *durum* (GCA_900231445.1 Svevo.v1); sequence IDs beginning with “WEW” are from the reference genome of *T. dicoccoides* wild emmer (GCA_002162155.2 WEW v2.0); sequence IDs beginning with “Hv” are from the reference genome of Barley (GCA_901482405.1_ Morex_v1.0,); sequence IDs beginning with “Bd” are from the reference genome of *Brachypodium* (GCF_000005505.3 *Brachypodium distachyon* v3.0); and sequence IDs beginning with “TA” are *Lr42* alleles from the *Ae. tauschii* accessions. Source data are provided as a Source Data file.


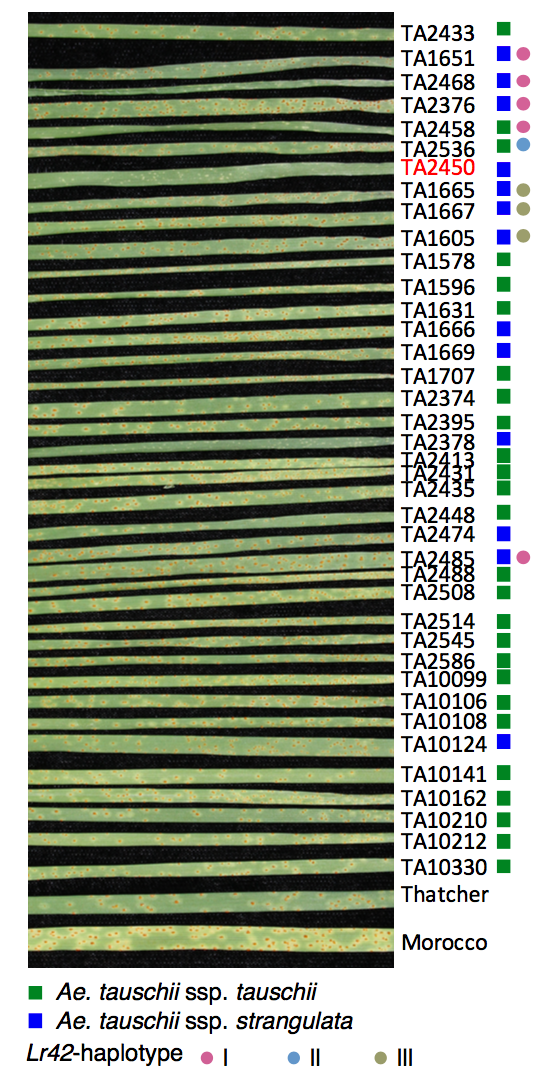


**Supplementary Fig. 13: Phenotype of *Ae. tauschii* minicore lines.** In total, 37 *Ae. tauschii* lines from the minicore set and the control lines TA2450, TA2433, Thatcher and Morocco were inoculated with race PNMRJ. Infection types of the *Ae. tauschii* minicore lines were presented in **Supplementary Date 3**. Solid colored circles represent *Lr42* haplotypes. Colored squares indicate lineages of accessions.


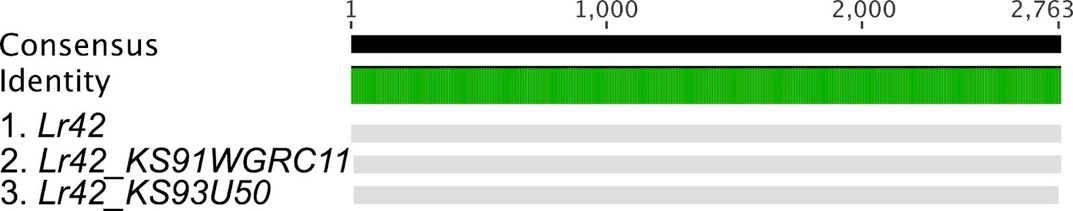


**Supplementary Fig. 14: Wheat accessions KS91WGRC11 and KS93U50 carry *Lr42*.** *Lr42* from the wheat line KS91WGRC11 and KS93U50 was amplified and sequenced. The multiple alignment was performed using ClustalW in Geneious. No vertical lines in three gray rectangles indicate the identity of the three sequences.

**
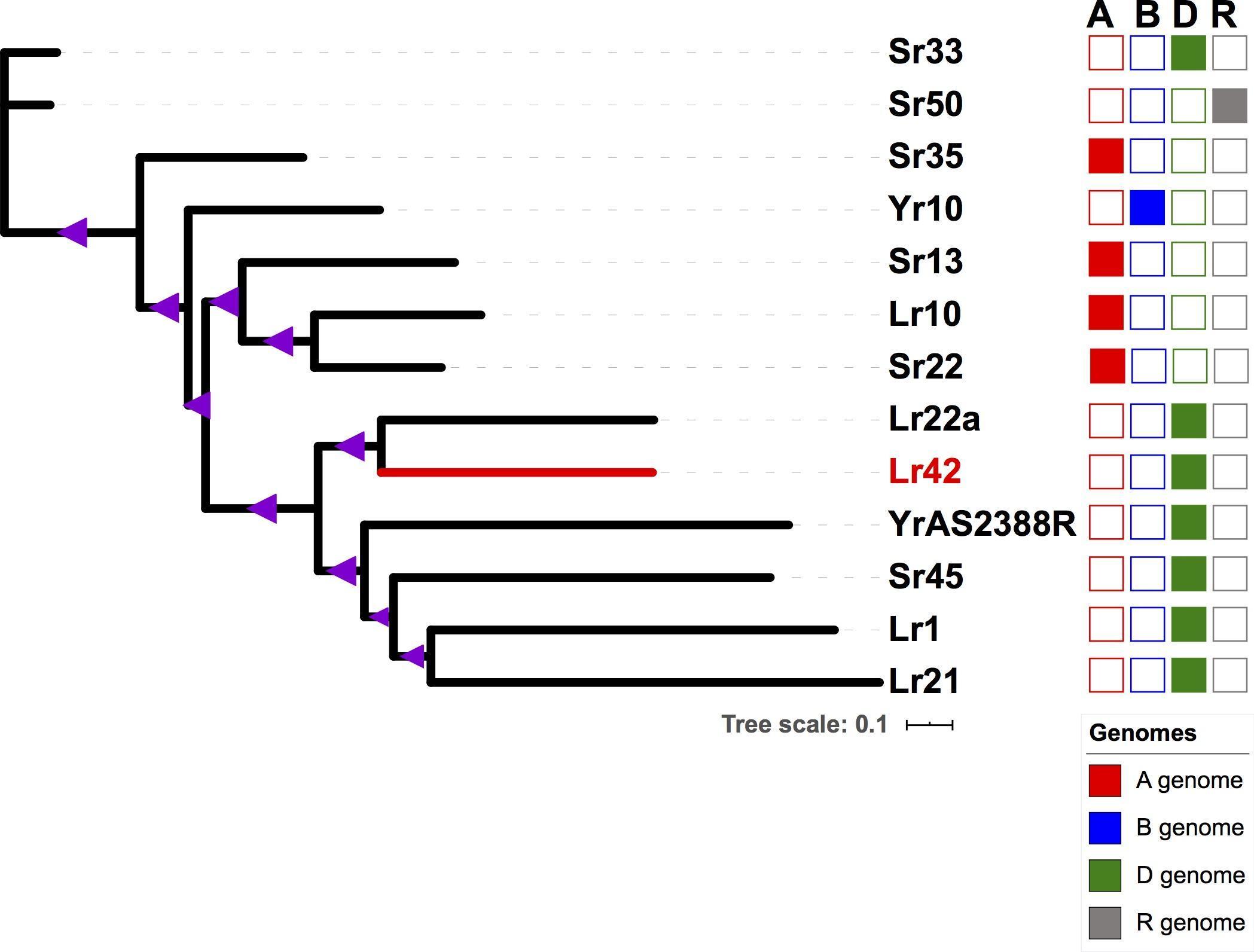
**

**Supplementary Fig. 15: Phylogenetic tree of cloned wheat rust resistance NLR proteins.** Multiple alignments were performed using ClustalW implemented in the Geneious software. The bootstrap values from 56% to 100% are indicated by purple triangles from small to large sizes. Lr22a has the highest identity with Lr42 (31.6%). The genes and their chromosomal locations are: *Lr1* (5DL), *Lr10* (1AS), *Lr21* (1DS), *Lr22a* (2D), *Lr42* (1DS), *Sr13* (R1 haplotype)(6A), *Sr22* (7A), *Sr33* (1DS), *Sr35* (3AL), *Sr45* (1D), *Sr50* (1RS), *YrAS2388R* (4DS), and *Yr10* (1BS).

**Supplementary Table 1: Avirulence/virulence specificity of races of *Puccinia triticina* used in this study**

| Race | Avirulent | Virulent |
| --- | --- | --- |
| PNMRJ | *Lr2a, Lr11, Lr14a, Lr16, Lr17, Lr21, Lr26, Lr42* | *Lr1, Lr2c, Lr3, Lr3ka, Lr9, Lr10, Lr18, Lr24, Lr28, Lr30, Lr39, LrB* |
| TFBJG | *Lr3ka, Lr9, Lr11, Lr16, Lr17, Lr18, Lr21, Lr30, Lr39, Lr42, LrB* | *Lr1, Lr2a, Lr2c, Lr3, Lr10, Lr14a, Lr24, Lr26, Lr28* |
| MBJ/SP | *Lr2a, Lr2b, Lr2c, Lr3ka, Lr9, Lr16, Lr18, Lr19, Lr21, Lr24, Lr25,* (*Lr26*)*, Lr28, Lr29, Lr30, Lr32, Lr33, Lr36, Lr42* | *Lr1, Lr3, Lr3bg, Lr10, Lr11, Lr13, Lr15, Lr17, Lr20, Lr23, Lr27+Lr31* |

**Supplementary Table 2: List of *Lr42*-specific GBS tags**

| **Order** | **Sequence (5'-3')** |
| --- | --- |
| 1 | TGCAGTCACATGCATGTGCACCCAGTGGCAGTCGGTTCTCTCCCTTAATCTGCTCCCTCTAAAC |
| 2 | TGCAGCTATCCTTAGCATGTACGTATGTTTCATCTCAAAAAGAAAAAAAGAGCTATCCTTAGCA |
| 3 | TGCAGTTGTTTTTTTGAGATGGAGCGCTATTAGATCTAATCCATTAATTACGTTTTTTAAGTTA |
| 4 | TGCAGTGATCCGATCCAGGTGTTTGAGGAAGCAGCGCGAGTGTTCAATGATGACTACTTCCTCA |
| 5 | TGCAGCTGGGTTTCGATTTTTTGGAGCTTGGCTGCCAGGCGGCGCCAGGTCTTGATATGCTTGA |
| 6 | TGCAGATGTTCTGCAAATTATAAACTACTCACTATACCATGAAAAAATTTAGGGGGGCACCATA |
| 7 | TGCAGGACTAGCTAGGTACGAAGAAGCAATTGTTTACCTGGTGCCGCCAGAGGGCAGGATGATT |
| 8 | TGCAGGCAGGTTGACGAGGGCATCTACTGTACACAAGGCCCGCAAATCAAATCAAGTCCAATTC |
| 9 | TGCAGTTGAAGGGCACAATTTTTTTGTTGGAGATTATAGTACGATAAATTCAGCTTGTACTCTA |
| 10 | TGCAGCACATTGACCTAATATACGAGGAACAAATATAGGAAGCTATTTATGCCTTCCAGATTCA |
| 11 | TGCAGGTGGTTGAGTATGCATCCCCAGCACTAACCCCAGCCCCAACCACACATGATACCTGTAG |
| 12 | TGCAGCTAGTTAAGTAACATTAGTAGGTACGCCTTTAATTTCTTGTTGAATACTCGCAAGTTTA |
| 13 | TGCAGGGTGTGTAATTTGAACAAAACCACGACGAGTAATTTAGAACGGAGGGAGTATATCATAT |
| 14 | TGCAGATCGATGGTGAGTGCGCCGACCGAGAGAAGGAAGGAGCTGCGGGGGATGCGGGGACGAG |

**Supplementary Table 3: Yield traits of wheat lines with and without *Lr42***

| Trait | number of *Lr42*+ | number of *Lr42*- | effect^*^ | p-value^†^ |
| --- | --- | --- | --- | --- |
| Test weight | 105 | 146 | 0.3 | 0.24 |
| Grain yield Severe drought | 105 | 146 | 0.21 | 0.282 |
| Thousand Kernel weight | 105 | 146 | 0.79 | 0.292 |
| Grain yield Optimum irrigation Bed planting | 105 | 146 | -0.09 | 0.414 |
| Grain yield Drought | 105 | 146 | 0.09 | 0.521 |
| Grain yield Optimum irrigation Flat planting | 105 | 146 | -0.05 | 0.614 |
| Grain yield Late-sown heat stress | 105 | 146 | -0.04 | 0.77 |

^*^ mean difference between *Lr42*+ and *Lr42*- lines

^†^ p-values from two-side t-tests without a multiple test correction

**Supplementary Table 4: MADA motif sequences of known wheat rust resistance NLRs**

| **NLR/motif**  **_name** | **Motif sequence**^%^ | | | | | | | | | | | | | | | | | | | | | | | | | | | | | | | | | |
| --- | --- | --- | --- | --- | --- | --- | --- | --- | --- | --- | --- | --- | --- | --- | --- | --- | --- | --- | --- | --- | --- | --- | --- | --- | --- | --- | --- | --- | --- | --- | --- | --- | --- | --- |
|  | **1** | 2 | 3 | 4 | 5 | 6 | 7 | 8 | 9 | 10 | | 11 | | 12 | | 13 | | 14 | | 15 | | 16 | | 17 | | 18 | | 19 | | 20 | | 21 | |  |
| **MADA** | **M** | **A** | **D** | **A** | **x** | V | **S** | **F** | **x** | **V** | | **x** | | **K** | | **L** | | **x** | | **x** | | **L** | | **L** | | **x** | | **x** | | **E** | | **x** | |  |
| Lr42 | M | A | E | A | V | V | G | Q | L | V | | V | | T | | L | | G | | E | | A | | L | | A | | K | | E | | A | |  |
| Lr22a | M | A | E | A | A | L | L | L | V | T | | T | | K | | I | | G | | K | | A | | V | | A | | T | | E | | T | |  |
| Lr1 | M | A | A | A | L | G | S | A | A | T | | L | | L | | G | | K | | V | | F | | T | | M | | L | | S | | A | |  |
| Lr21 | M | A | T | A | W | D | V | A | S | V | | G | | W | | S | | M | | V | | V | | L | | G | | W | | L | | V | |  |
| Lr10 | M | A | P | C | L | V | S | A | S | T | | G | | A | | M | | G | | S | | L | | L | | T | | K | | L | | E | |  |
| Sr45 | M | A | E | F | V | V | R | P | L | V | | S | | T | | L | | M | | N | | T | | A | | S | | S | | Y | | L | |  |
| Sr13 | M | A | E | F | V | V | R | P | L | V | | S | | T | | L | | M | | N | | T | | A | | S | | S | | Y | | L | |  |
| Sr22 | M | A | E | V | L | V | S | A | S | T | | G | | A | | M | | G | | S | | L | | L | | R | | K | | L | | G | |  |
| YrAS2388R | M | A | G | V | L | D | A | L | A | S | | Y | | V | | T | | N | | M | | L | | T | | E | | M | | A | | K | |  |
| Sr33 | M | D | I | V | T | G | A | I | A | K | | L | | I | | P | | K | | L | | G | | E | | L | | L | | V | | G | |  |
| Sr35 | M | E | I | A | M | G | A | I | G | S | | L | | L | | P | | K | | L | | G | | E | | L | | L | | I | | G | |  |
| Yr10 | M | E | V | V | T | G | A | M | S | T | | L | | L | | P | | L | | L | | G | | D | | L | | L | | K | | E | |  |
| Sr50 | M | N | I | V | T | G | A | M | G | S | | L | | I | | P | | K | | L | | G | | E | | L | | L | | M | | D | |  |
| ^%^ First 21 aa of each NLR protein were listed. | | | | | | | | | | |  | |  | |  | |  | |  | |  | |  | |  | |  | |  | |  | |  | |

| Country | Name | Pedigree |
| --- | --- | --- |
| Afghanistan | Wafer 15 | BABAX/LR42//BABAX*2/3/TUKURU |
| Afghanistan | Koshan 09 | BABAX/LR42//BABAX*2/3/VIVITSI (=QUAIU) |
| Ethiopia | Gambo | BABAX/LR42//BABAX*2/3/VIVITSI (=QUAIU) |
| Iran | Aftab | THELIN/3/BABAX/LR42//BABAX/4/BABAX/LR42//BABAX |
| Kenya | Kenya Peacock | QUAIU/3/PGO/SERI/BAV92 |
| Mexico | RSM Norman F2008 | BABAX/LR42//BABAX |
| Nigeria | Norman | BABAX/LR42//BABAX |
| Rwanda | Reberaho | BABAX/LR42//BABAX*2/4/SNI/TRAP#1/3/KAUZ*2/TRAP//KAUZ |
| Rwanda | Rengerabana | BABAX/LR42//BABAX*2/3/TUKURU |
| Turkey | Ekinoks | BABAX/LR42//BABAX*2/3/VIVITSI (=QUAIU) |
| Kenya | Robin | BABAX/LR42//BABAX*2/3/TUKURU |

**Supplementary Table 5: CIMMYT varieties developed from *Lr42* wheat lines**
