# Supplementary material for: Cloning of the broadly effective wheat leaf rust resistance gene Lr42 transferred from Aegilops tauschii
Source: Nat Commun. 2022 Jun 1;13:3044. doi: 10.1038/s41467-022-30784-9 (PMC9160033; doi:10.1038/s41467-022-30784-9)
Supplement: Supplementary file 3 — Description of Additional Supplementary Files [file 41467_2022_30784_MOESM3_ESM.pdf]

Description of Additional Supplementary Files

File Name: Supplementary Data 1
Description: KASP markers used for fine mapping in *Ae. tauschii*

File Name: Supplementary Data 2
Description: KASP markers useful for marker-assisted selection in hexaploid wheat

File Name: Supplementary Data 3
Description: List of *Ae. tauschii* accessions used in the study

File Name: Supplementary Data 4
Description: Clusters of *Lr42* homologs

File Name: Supplementary Data 5
Description: Presence (1) or absence (0) of the Lr42 segment in CIMMYT lines based on having at least five *Lr42*-specific GBS tags or none

File Name: Supplementary Data 6
Description: The resutls of KASP assays in CIMMITY breeding lines with Lr42- pD1 and Lr42-pD2

File Name: Supplementary Data 7
Description: *Lr42* marker validation using Hard Winter Wheat Association Mapping Panel (HWWAMP) plus known positive controls.

File Name: Supplementary Data 8
Description: List of primers and their sequences
