# Supplementary figures and images for: Cloning of the broadly effective wheat leaf rust resistance gene Lr42 transferred from Aegilops tauschii
Source: Nat Commun. 2022 Jun 1;13:3044. doi: 10.1038/s41467-022-30784-9 (PMC9160033; doi:10.1038/s41467-022-30784-9)

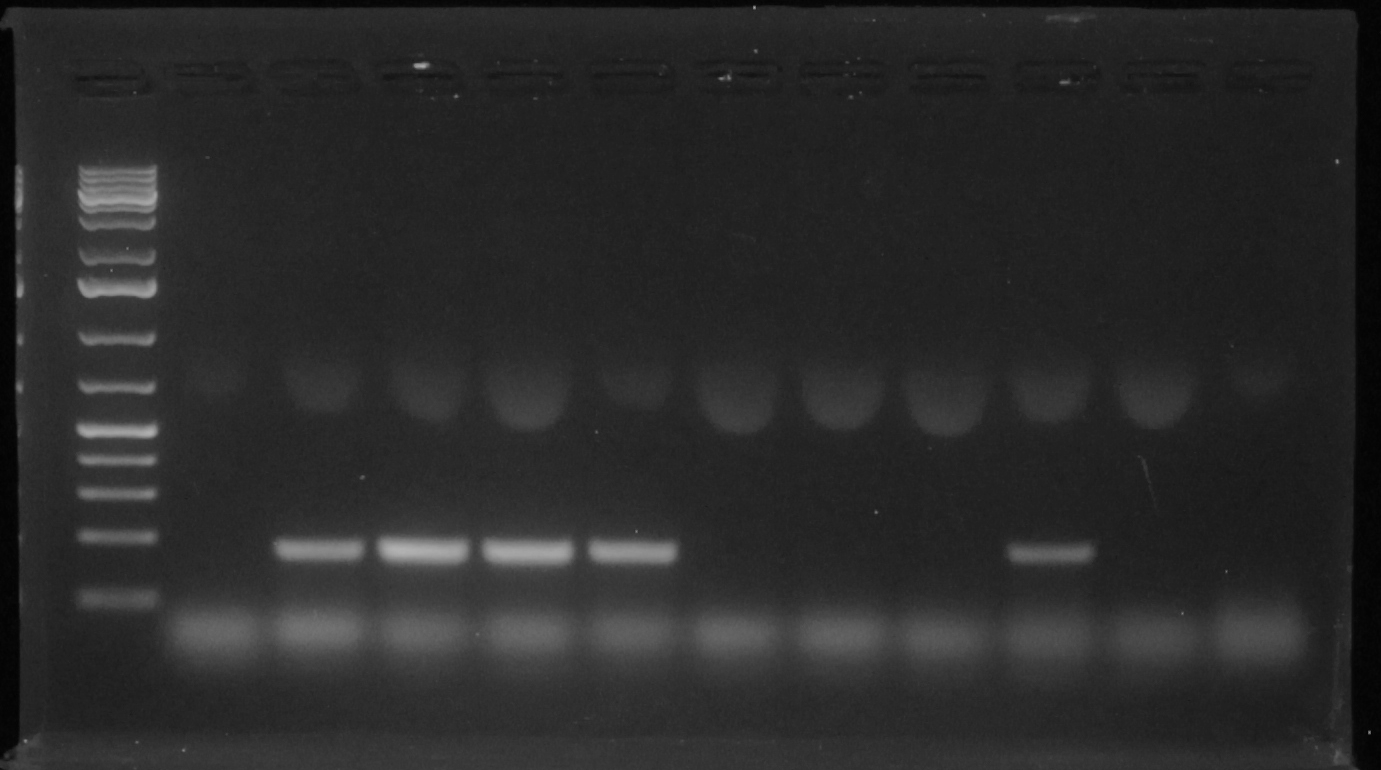

Supplement: Supplementary file 13 — Source Data [file 41467_2022_30784_MOESM13_ESM.zip › 08_SourceData/SourceData3_Figure2b_Lr42R.TIF]

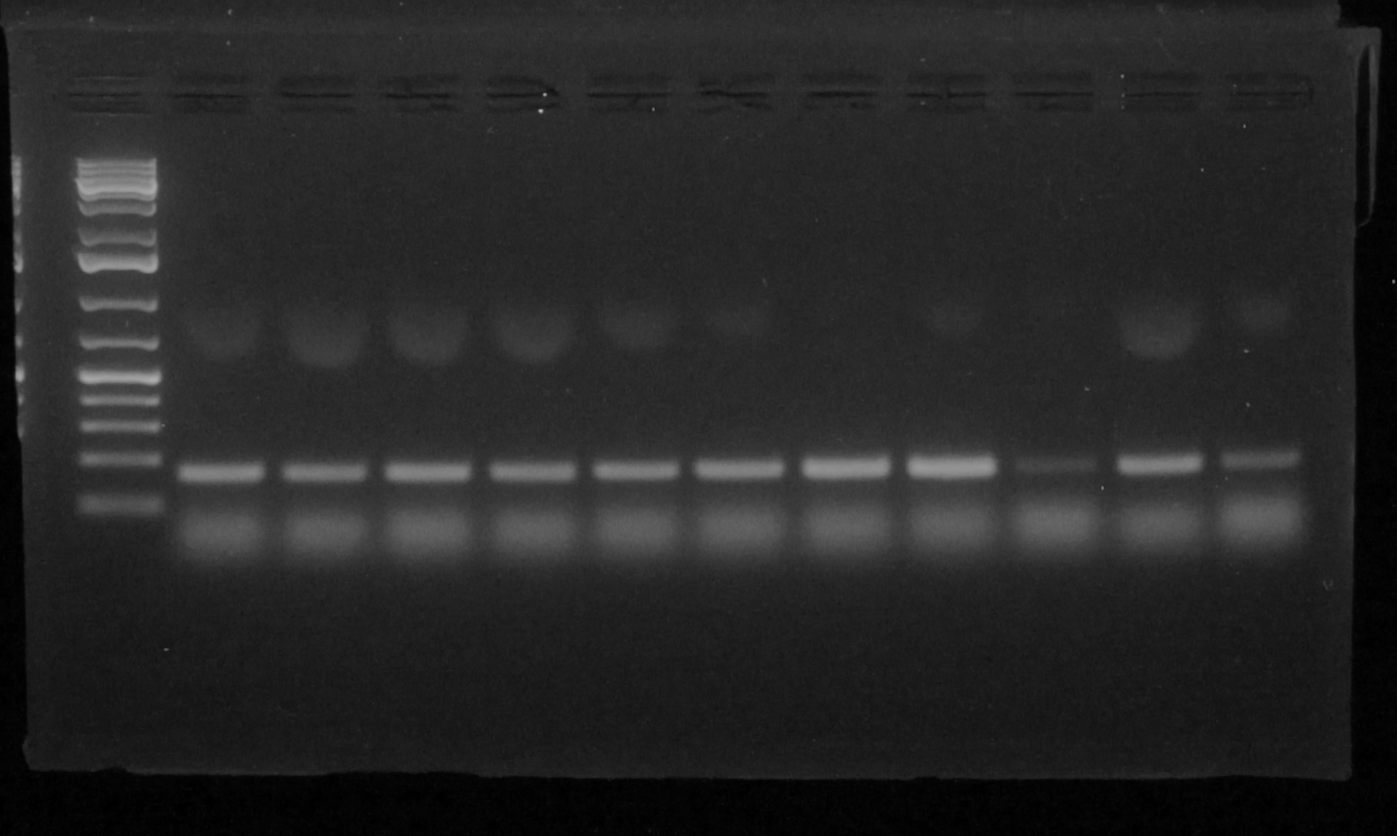

Supplement: Supplementary file 13 — Source Data [file 41467_2022_30784_MOESM13_ESM.zip › 08_SourceData/SourceData4_Figure2b_actin.TIF]

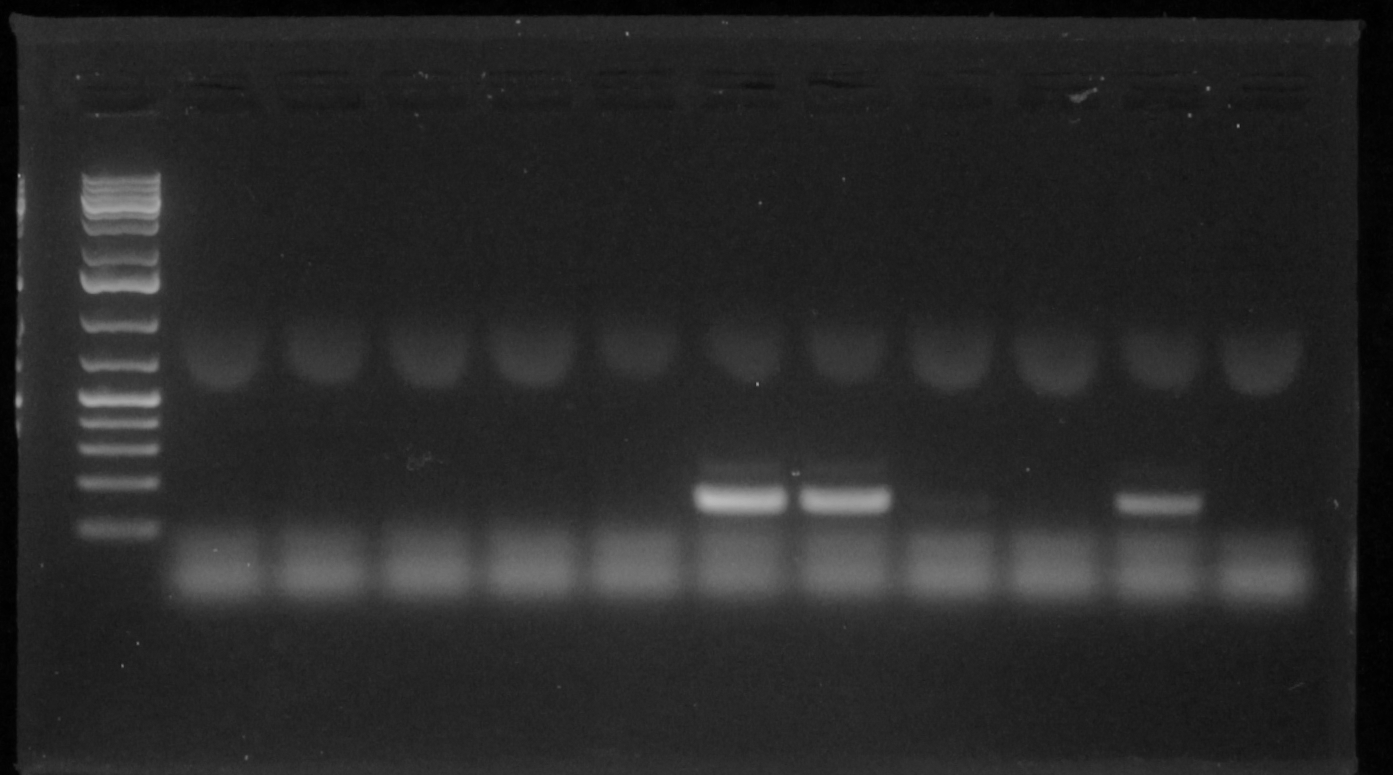

Supplement: Supplementary file 13 — Source Data [file 41467_2022_30784_MOESM13_ESM.zip › 08_SourceData/SourceData5_Figure2b_lr42.TIF]
